# Unboxing Industry-Standard AI Models for Male Fertility Prediction with SHAP

**DOI:** 10.3390/healthcare11070929

**Published:** 2023-03-23

**Authors:** Debasmita GhoshRoy, Parvez Ahmad Alvi, KC Santosh

**Affiliations:** 1 School of Automation, Banasthali Vidyapith, Tonk 304022, Rajasthan, India; debasmitaghoshroy@banasthali.in; 2 Department of Physics, Banasthali Vidyapith, Tonk 304022, Rajasthan, India; aparvez@banasthali.in; 3 Applied AI Research Lab, Vermillion, SD 57069, USA; 4 Department of Computer Science, University of South Dakota, Vermillion, SD 57069, USA

**Keywords:** male infertility, oversampling, explainable artificial intelligence (XAI), Shapley additive explanations (SHAP)

## Abstract

Infertility is a social stigma for individuals, and male factors cause approximately 30% of infertility. Despite this, male infertility is underrecognized and underrepresented as a disease. According to the World Health Organization (WHO), changes in lifestyle and environmental factors are the prime reasons for the declining rate of male fertility. Artificial intelligence (AI)/machine learning (ML) models have become an effective solution for early fertility detection. Seven industry-standard ML models are used: support vector machine, random forest (RF), decision tree, logistic regression, naïve bayes, adaboost, and multi-layer perception to detect male fertility. Shapley additive explanations (SHAP) are vital tools that examine the feature’s impact on each model’s decision making. On these, we perform a comprehensive comparative study to identify good and poor classification models. While dealing with the all-above-mentioned models, the RF model achieves an optimal accuracy and area under curve (AUC) of 90.47% and 99.98%, respectively, by considering five-fold cross-validation (CV) with the balanced dataset. Furthermore, we provide the SHAP explanations of existing models that attain good and poor performance. The findings of this study show that decision making (based on ML models) with SHAP provides thorough explanations for detecting male fertility, as well as a reference for clinicians for further treatment planning.

## 1. Introduction

Male reproduction is a complex biological process, and a rising trend in male factor infertility has been observed in the last few years. According to the WHO, nearly 15% of couples worldwide live with infertility [1]. The male is found to be the sole cause in 20% of these cases and a partial contributor in 30% to 40% [2]. Many clinical reasons are associated with male infertility, and plenty of documents have highlighted that lifestyle and environmental factors potentially impact sperm parameters and degrade semen quality. These factors include the use of tobacco, alcohol, and drugs; psychological stress; obesity; lack of sleep; and environmental factors such as air pollutants and heavy metals. Moreover, a sedentary lifestyle with more than 4 h of daily sitting is significantly associated with a higher proportion of immotile sperm [3,4,5]. Hence, male infertility can be prevented by modifying these factors, which helps to reduce the overall incidence of male infertility. However, a healthy lifestyle and environment are suitable for general health. 

Artificial intelligence (AI) has been widely used in clinical sectors in this modern era, and reproduction medicine is no exception [6]. Prediction and treatment recommendation are the two most essential applications in reproductive medicine. Additionally, ongoing studies are being conducted to enhance future directions. Many studies have considered ML-based solutions for detecting male fertility. Gil et al. [7] conducted the first study on male fertility analysis using support vector machine (SVM), multi-layer perceptron (MLP), and decision tree (DT) classifiers. The accuracy of SVM and MLP in detecting sperm concentration and morphology was 86% and 69%, respectively. Sahoo and Kumar [8] selected five classifiers: DT, MLP, SVM, SVM-particle swarm optimization (PSO), and naïve bayes (NB). Of all, SVM-PSO outperformed with an accuracy of 94%. Bidgaoli et al. [9] selected four AI tools: optimized MLP, NB, DT, and SVM. Of all, optimized MLP provided the maximum outcome of 93.3%. Girela et al. [10] opted for MLP, and 90% and 82% accuracy were achieved for sperm concentration and sperm motility, respectively. Soltanzadeh et al. [11] selected NB, neural network, logistic regression (LR), and fuzzy C-means. Of all, NB provided the best outcome, with an AUC of 0.779. Rhemimet et al. [12] utilized DT and NB, and the maximum accuracy of 88.63% was achieved via NB. Candemir et al. [13] used MLP, SVM, DT, and FRBF. Of all, FRBF achieved the best accuracy of 90%. Simfukwe et al. [14] employed artificial neural network (ANN) and NB, and both classifiers obtained an accuracy of 97% in the training phase. Ahmed and Imtiaz [15] selected NB, and 87.75% accuracy was reported. Five different classifiers were chosen by Engy et al. [16], including ANN, ANN-genetic algorithm (GA), DT, SVM, and ANN-SWA. The maximum accuracy was achieved by ANN-SWA (99.96%). Mendoza-Palechor et al. [17] used supervised and unsupervised learning techniques to predict male fertility status. The K-means algorithm is used for clustering and J48, SMO, NB, and lazy IBK are provided as the classification report. The NB model outperforms the others with TP and FP rates of 98.4% and 1.50%, respectively. Ma et al. [18] used three classifiers: SVM, adaboost (ADA), and back propagation neural networks (BPNN). Of all, ADA performed best, with an accuracy of 95.1%. Dash and Ray [19] selected eight classifiers: soft voting, DT, NB, LR, DT, DT bagged, RF, and extra tree (ET). The maximum accuracy of 90.02% was achieved via ET. Ghosh Roy and Alvi [20] proposed a model based on K-nearest neighbor classifiers, and 90% accuracy was reported. Yibre and Kocer [21] used a feed-forward neural network (FFNN), and 97.50% accuracy was reported. Ghosh Roy and P.A. Alvi et al. [22] proposed an explainable model to detect male fertility. The authors used the xgboost (XGB) classifier, and 93.22% mean accuracy was reported with five-fold CV.

Although existing AI models performed well, most of them only discuss the model’s accuracy. Consequently, those models cannot explain how and why any decision has been made. As a result, these AI models are treated as a black box and their usage is still limited in male fertility detection. In this study, we use existing ML techniques to predict male fertility with proper explanation. It helps to understand how inputs and outputs are associated; thus, the prediction results enhance accountability, explainability, and transparency. As a result, the users (clinicians) can easily understand the prediction process and verify the results given by AI models. In this way, we can commercialize the existing AI systems in male fertility analysis. Additionally, these models bring a lot of improvement in primary care, which may pave the way for more accurate diagnosis. Furthermore, skewness in the dataset and model validation are crucial issues that cannot be neglected when designing effective AI models. Therefore, we integrate these three aspects, which help commercialize the existing AI systems in male fertility detection. The critical contributions of this paper are:

- Seven industry-standard ML models are analyzed for male fertility detection.
- To assess the robustness and stability of each model, we employ sampling and two different CV techniques on each AI model.
- XAI is used to explain the performance of each good and poor model and uncover the black box.

## 2. Problem Background

The problem-oriented literature is discussed in the coming subsections that help to understand the background of the research motivation by considering class imbalance learning, sampling techniques, classifier selection, and validation schemes.

### 2.1. Class Imbalance Learning

In AI, it is crucial to construct a practical AI model over an imbalanced class dataset due to characteristics of small sample size, class overlapping, and small disjuncts [23,24]. 

#### 2.1.1. Small Sample Size

With fewer samples and unequal distribution of majority and minority classes, it is not easy to detect and prevent the learning system from capturing their characteristics and hindering the generalization capability of AI models. This situation becomes very challenging when the class imbalance ratio is large.

#### 2.1.2. Class Overlapping

It is the most common problem of dealing with an imbalanced dataset. It has a higher negative impact on the performance of AI models. In this problem, the region of data space contains a similar quantity of training data from each class, leading to the development of a learning model with almost the exact prior probabilities in this overlapping area. As a result, it is very tough to make a distinction between the two classes.

#### 2.1.3. Small Disjuncts

This occurs when the concept represented by the minority class is formed by sub-concepts or sub-clusters, which have low coverage between them in the data space. As an outcome, it is difficult to know whether these examples represent an actual sub-concept. Thus, AI systems are typically biased towards classifying large juncts and tend to overfit and misclassify the cases in small disjuncts.

### 2.2. Sampling Approaches

The sampling technique is a probable solution to deal with imbalanced dataset-related issues. The most common approaches are undersampling and oversampling. Researchers have selected a specific approach to improve the model performance depending on dataset distribution. In oversampling, synthetic samples are generated from the minority class. Similarly, undersampling eliminates the majority samples from the minority class. Over the years, various sampling strategies have been implemented with popular AI algorithms.

Many samplers such as SMOTE, ADASYN, SLSMOTE, DBSMOTE, CUS, DROS, and ESLSMOTE are available to handle this imbalanced class issue [25,26]. Apart from these conventional techniques, the combination sampling approach is applied occasionally, improving the performance compared with the techniques performed in isolation. Moreover, in disease classification, the synthetic minority oversampling technique (SMOTE) is widely used to balance the dataset, improving the model performance. The growing body of evidence suggests that data balancing is essential in effective AI model design.

### 2.3. Classifier Selection

Classifier or algorithm selection is vital in constructing a predictive model based on features. Appropriate and suitable classifier selection help to achieve better classification performance. The main aim is to study the relationship between input variables and a target variable (binary) of interest. For example, whether the person is normal or diseased. The choice of classifiers is considered based on the dataset size and features. If the target label is categorical and labeled, then classification algorithms are the possible solution to design the model. On the other hand, if the dataset is unlabeled, clustering algorithms need to be applied. The dataset size is large, so the choice of classifier probably has little effect on model performance. In contrast, ML algorithms perform better if the dataset size is limited. Additionally, based on the previous experiment, we can easily select the classifiers. 

### 2.4. Validation Schemes

Every model has advantages and disadvantages, and some classifiers have a higher tolerance for small datasets, whereas others perform well with large datasets. For this reason, each model prediction result is different with the application of the same inputs. Therefore, a validation protocol is fundamental to understanding the model performance to unseen data [27]. This process comes into the picture when model development is completed. Consequently, the validation process is a part of the model design. In this stage, the focus is on both schemes, such as statistical and business metrics, which help to conclude model reliability and relevance. It is crucial that the developed model precisely replicates the result in real-time applications. A few more advantages come from validations: it can prevent overfitting and underfitting, increase scalability, enhance model quality, and reduce cost. 

### 2.5. XAI Tools

Presently, XAI is gaining popularity, especially in the medical sector. It is a set of tools and frameworks that helps to understand and interpret AI systems’ predictions. The most widely used XAI tool for clinical analysis is SHAP, which uses the Shapley value from the concept of cooperative game theory [28]. The Shapley value is defined as the average of all marginal contributions to all possible coalitions. The computation time increases exponentially with the number of features. One solution to keep the computation time manageable is to compute contributions for only a few samples of the possible coalitions. The SHAP is applicable for both classification and regression problems. 

## 3. Material and Methods

**Figure 1:**
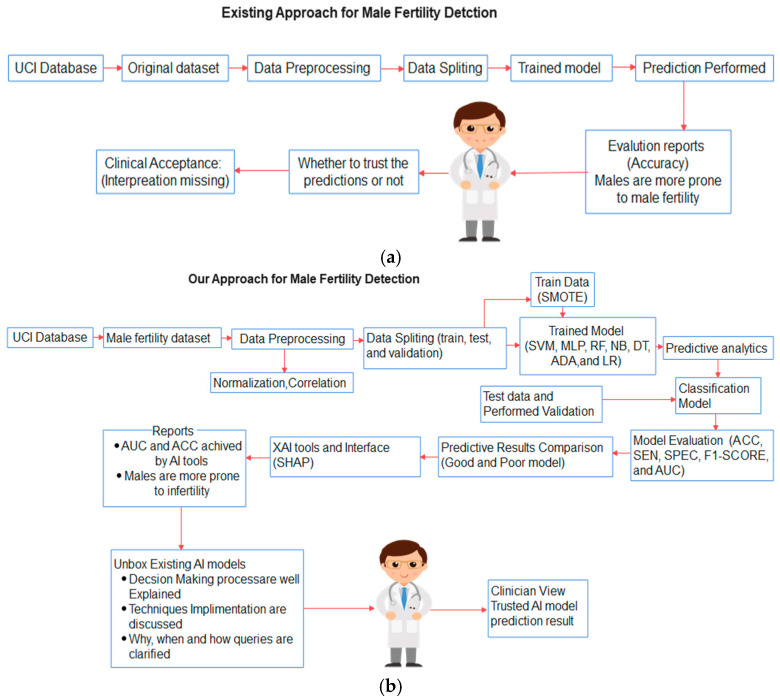
(**a**) ML approach without XAI. (**b**) ML approach with XAI.

This section describes sufficient details of the conceptual framework, including the methods used in this experiment. Figure 1a,b illustrates the schematic presentation of existing vs. our approach to detect male fertility. Figure 1a represents the existing or conventional approach, where the clinician is not confident to accept the outcome of ML models due to the lack of interpretation. As a user, it is also tough to understand the workflow and algorithmic approaches adopted by ML. For this reason, clinicians are not sure about the prediction process of ML models and real-time implementation gets hampered. Similarly, Figure 1b replicates the upgraded version of the existing approach integrated with XAI. The main three key queries, why, when, and how, are delivered via explainable AI with interpretability. Applying XAI on existing black boxes is necessary to explain the result shown by ML and interpret how the decision has been made. As a result, the clinician can trust the decision-making process with a proper explanation of ML models. In addition, the SMOTE technique is used to balance the dataset distribution, which can prevent the biasness of a model towards one class. Moreover, our approach provides a proper explanation of the prediction decisions and references to clinicians for further treatment planning. Hence, commercialization of ML models is possible along with real-time implementation.

### 3.1. Data Source and Information

In this study, we collected the data from the UCI repository. The dataset covers 9 input features, including environmental and lifestyle factors followed by WHO criteria. A total of 100 samples are present; among them, 88 samples are considered as normal seminal quality and 12 samples belong to altered seminal quality. The imbalance ratio of this dataset is 1:7, and the detailing of features is represented in Table 1.

### 3.2. Analysis of Dataset

Data analysis is required, which includes visualization, statistical overview (mean, median, and mode), and measure of relationship (correlation). 

#### 3.2.1. Statistical Overview

The mean, median, and mode are the most useful measure to identify the sample pattern. The mean and median are helpful when data is relatively homogenous and heterogenous, and the mode is useful when one value occurs frequently. In this study, the dataset contains 10 features, including the target label. The input data is converted into a range of normalization with all information stored in it. The normalized values of the dataset are depicted in Table 1. Conversely, Table 2 presents the statistical overview of different features related to the numerical and categorical male fertility dataset.

#### 3.2.2. Measure of Relationship

**Figure 2:**
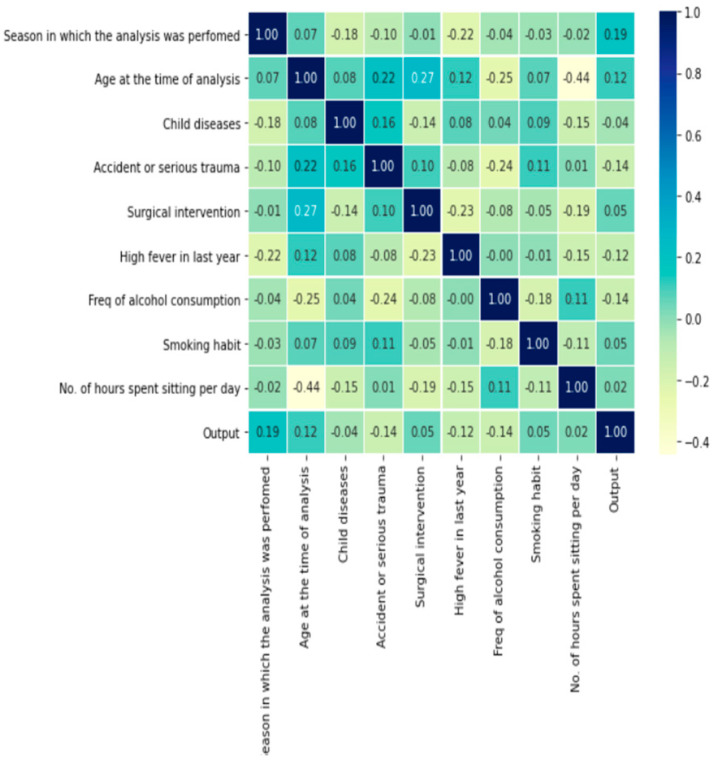
Measure of relationship using correlation.

A clear visualization and correlation process are applied to understand the data pattern. A correlation matrix is a table that depicts the correlation coefficient between two or more variables. We have used Pearson’s coefficient for this dataset, and the correlation size can measure each feature’s dependency. The expression of Pearson’s correlation is expressed in $r=\frac{\sum (\;x_{i}-\bar{x)}\;\left(y_{i}-\bar{y}\;\right)}{\sqrt{\sum {\left(x_{i}-\bar{x}\;\right)}^{2}\;\sum {\left(y_{i}-\bar{y}\right)}^{2}\;}}$ , and r is a co-relation coefficient, $x_{i}$ represents the distinct values of x present in sample x, and $y_{i}$ represents the distinct values of y in a sample. x and y are averaged values of x and y, respectively. Figure 2 represents the relationship of each feature, where a blue color indicates a positive correction and yellow signifies a negative one. For a better understanding, the values are listed in Table 3.

### 3.3. Sampling Technique

In this study, we have used the SMOTE technique, which represents the baseline over the sampling approach [29]. In this method, the main aim is to reduce the majority class samples and increase the minority class, i.e., the binary class distribution is 1:1. The mathematical expression of SMOTE is presented in Equation (1)

(1) $$\mathrm{Total}\;\mathrm{instances}=p_{c}+k_{c}+k_{'c}$$

where $p_{c}$ and $k_{c}$ represent the number of majority and minority class instances.

Now, after the application of the oversampling technique, the total data is computed and is presented in Equation (2)

(2) $$k_{'c}=\left(1-z_{c}\right)\times k_{c}$$

where the imbalance ratio of $p_{c}\;and\;k_{c}$ is represented as $z_{c}.$ After applying SMOTE to our training dataset, the original amount of data is raised by 600%. The description is summarized in Table 4.

### 3.4. Overview of Classification Models

We have employed seven industry-standard machine learning classifiers: SVM, RF, DT, LR, NB, ADA, and MLP. These models are effective in classification tasks with limited samples.

#### 3.4.1. SVM

This algorithm was first invented by Vapnik [30] and can provide better classification performances than other classification techniques. The main objective of this classification technique is that it separates a set of training vectors for two different classes $\left(x_{1},y_{1}\right),\left(x_{2},y_{2}\right),\ldots ,\left(x_{m},y_{m}\right),\;$ where $x_{i}\epsilon R^{d}$ denotes vectors in a d-dimensional feature space and $y_{i}\;\epsilon \;\left\{-1,+1\right\}$ is a class label. The SVM model is generated by mapping the input vectors onto a new higher-dimensional feature space. Then, an optimal separating hyperplane in the new feature space is constructed using the kernel function. This kernel function is the product of input vectors. Three different types of kernels, such as linear, RBF, and polynomial, are available. A linear kernel is mostly used as per the literature, but there is no formal way to decide the best kernel function for a specific area. 

#### 3.4.2. RF

It is a popular classifier in many clinical settings that was initially proposed by Tin Kam Ho [31]. After that, Amit and Geman [32] and later Brieman [33] invented the integral form known as RF. It is an ensemble learner based on a decision tree. For classification tasks, the output of the RF is the class selected by most trees. It adopted the bootstrap resampling approach to repeatedly randomly select samples from the original training sample set of N as the training set and the remaining samples as the test set. 

#### 3.4.3. DT

Quinlan [34] invented this classifier, one of the oldest and most popular approaches. This classification technique describes the data points by a collection of attributes. These are shown as trees, with each node representing features and each offspring representing a possibility. Each leaf corresponds to a decision rule. Wide varieties of DT are available such as ID3, CART, C4.5, and MARS. In this study, we utilized ID3 with the following steps:

First, evaluate the entropy of each attribute in the dataset.

Divide the dataset into sub-categories by use of the property that gives the optimal information gain.

If the entropy value is zero, the corresponding node is considered a leaf node and no further splitting is required. Similarly, further splitting is required if the entropy value exceeds zero, as specified in the previous step.

#### 3.4.4. LR

It is the most popular choice for a data scientist and was invented by Berkson [35]. Logistic refers to the underlying logit function utilized to model the binary outcome. The statistical form of the binary logistic regression model as:

(3) $$P\;\left(y\right)=\frac{1}{1-e^{-\left(b_{0}+b_{1}x_{1}+b_{2}x_{2}\right)}}$$

where P (y) indicates the probability of one category of the dependent variable *y*, *b* is the co-efficient of the independent variables or predictors, and *x* is the independent variables. 

#### 3.4.5. NB

This algorithm was invented by Bayes [36] based on the assumption that a particular feature’s effect is independent of others’ features. The basic formula of naïve Bayes is:

(4) $$p\left(\frac{h}{D}\right)=\frac{p\left(\frac{D}{h}\right)*P\left(h\right)}{P\left(D\right)}\;\;$$

where *P(h)* is the earlier likelihood of the theory *h;*

*P(D)* is the likelihood of information or otherwise called earlier likelihood;

$p\left(\frac{h}{D}\right)$ is the likelihood of theory h in given information of *D*, the posterior likelihood;

$p\left(\frac{D}{h}\right)\;$ is the likelihood information when speculation *h* is a good, known posterior likelihood. 

#### 3.4.6. ADA

This algorithm was proposed by Freund and Schapire [37] and derived from the concept of boosting. The boosting approach initially entails converting weak and strong learners. The ADA classifier iteratively trains multiple learning classifiers using the same training dataset. After training the weak learners, they are combined to obtain a robust classifier. The ADA procedure involves selecting an appropriate weak learner and employing the same training dataset to train the weak learners iteratively to improve their performance. The sample weight and each weak learner’s weight are used to execute ADA. The method adjusts sample weight based on the weak classifier results, focusing on erroneously classified data. Subsequent base learns are trained with the adjusted samples. The final robust classifier is obtained by combining the output of the weak learners using a weighted sum [38]. 

#### 3.4.7. MLP

This algorithm was invented by Rosenblatt [39]; it is a complex function that accepts numerical input and produces numerical output. The architecture of MLP is made up of three layers: (1) the input layers, (2) the hidden layers, and (3) the output layers. Input layers take the raw input from the domain, whereas output layers make a prediction. Hidden layers are used to extract features between the input and output layers. The number of hidden layers and neurons is referred to as the hyper-parameters of the MLP. Typically, hidden and output neurons in the MLP networks deploy activation functions (f). The value of f is generally equal but depends on the prediction type; a different f has been used in many cases. Sigmoidal and ReLU are popularly chosen for f in neural networks. The output of the MLP network is determined by weights and bias as well as the inputs. In general, MLP is used to predict the target class in binary. For the MLP model, input and output values are used to determine a set of weights and bias values. As a result, MLP generates computed output that closely matches the known output. 

### 3.5. Validation Schemes and Performance Metrics

#### 3.5.1. Validation Schemes

Many types of CV are available in the literature; k-fold and hold-out CV are the most popular choices for researchers. An 80–20 splitting ratio is used in hold-out CV, where 80% of the data is for training and 20% is for testing [40]. In contrast, the K-fold CV dataset was randomly split into k groups. One group is used as the test data, and the rest is trained data. The process is repeated until each group is used as the test set. The value of k must be in a defined range and chosen under where the minimum error rate is found [41]. For example, if k = 5, the dataset is split into 5 groups and the model is trained and tested 5 times so that each group is used as the test set. After 5 separate trials of training and testing, the result was averaged over the 5 trials to access the model’s mean (and standard deviation) performance for the dataset. 

#### 3.5.2. Performance Metrics

Five different evaluation protocols are applied: (1) accuracy (ACC), (2) sensitivity (SEN), (3) specificity (SPEC), (4) F1-score, and (5) area under the curve (AUC). These metrics are computed as follows:

(5) $$\mathrm{ACC}=\left(t_{p}+t_{n}\right)/\left(t_{p}+t_{n}+f_{p}+f_{n}\right)$$

(6) $$\mathrm{SEN}=t_{p}/\left(t_{p}+f_{n}\right)$$

(7) $$\mathrm{SPEC}=t_{n}/\left(t_{n}+f_{p}\right)$$

(8) $$F1\text{-}score=2\left(\frac{PREC\times SEN}{PREC+SEN}\right)$$

(9) AUC=0.5×(1+TPR−FPR)

In this study, we are dealing with balanced and imbalanced datasets. Hence, accuracy is not a good metric to measure model performance. We are focused on sensitivity, specificity, and AUC, which is considered an important measure for clinical analysis, replicating the model’s robustness.

### 3.6. SHAP

The term SHAP refers to “SHapley Additive exPlanations.” It is a technique for determining a given factor’s influence on the dependent variable’s value. The essential idea is that a feature’s significance depends not only on that feature but on all the features in the dataset. SHAP uses combinatorial calculus to retrain the model through all possible combinations of features and measures the impact of each feature on the target variable (the SHAP value). An attribute’s significance can be measured by calculating its average absolute impact value against a target variable. Hence, the measurement of the SHAP value depends on the model [42]. The SHAP value $\emptyset_{i}$ of feature *i* is defined as:

(10) $$\emptyset_{i}=\frac{1}{\left|N\right|!}\sum_{s\subseteq Nleft\left\{i\right\}}\left|S\right|!\left(\left|N\right|-\left|S\right|-1\right)![f\left(S\cup \left\{j\right\}-f\left(s\right)\right].$$

The number of elements in a set is represented by |·|. The original feature set is denoted by *N*, whereas any feature subset is represented by *S*. *Nleft*{*i*} is a subset of all elements in the sequence before feature *i*. The output of AI models is represented by *f*(*s*) for the feature subset *S*.*f*(*S* ∪{i} − *f*(*s*) that represents the cumulative contribution value of feature *i*. 

From Equation (10), we observed that the SHAP value $\emptyset_{i}$ of feature *i* is obtained by averaging the contributions in all possible permutations of the unique collection. SHAP can accurately measure the impact of any feature [43,44]; hence, it is possible to utilize the SHAP value to calculate the significance of the feature. 

## 4. Results and Analysis

This section discusses the results and their analysis using numerical data and a visualization approach. Before the classification task, the dataset is divided into three parts: training, testing, and validation. For training, 65% of data are used, whereas 20% is used for testing and the remaining is used in validation. Two CV schemes are employed for model evaluation to avoid overfitting. The total result is discussed in four steps: (1) the models’ performance with the imbalanced dataset, (2) the models’ performance with the balanced dataset, (3) XAI explanation with the imbalanced dataset, and (4) XAI explanation with the balanced dataset.

### 4.1. Performance of the Models with the Imbalanced Dataset

The first step of our experiments deals with the imbalanced dataset. The experimental observations are listed in Table 5. On this dataset, the RF classifier achieves classifications for ACC, SEN, SPEC, F1-score, and AUC of 96.67%, 0.965, 0.965, 0.982, and 0.932, respectively, which is better than others. In contrast, DT attains the lowest classifications for ACC, SEN, SPEC, F1-score, and AUC of 80.00%, 0.956, 0.142, and 0.648, respectively. At the same time, SVM, LR, and ADA attained the same accuracy of 93.33% with different AUCs of 0.887, 0.862, and 0.908, respectively. All models are validated via hold-out CV.

We present the observations using a five-fold CV scheme on the same dataset. The experimental results are documented in Table 6. In this case, standard deviation (STD) is reported in all cases, and the low value signifies the statistical robustness of each model. We identify SVM and LR attain the same classification ACC of 88.00% but the AUCs are 0.959 and 0.910, respectively. Similarly, NB achieves a low ACC and AUC of 67.00% and 0.500, respectively, among all classifiers. For LR and SVM, the value of STD is 0.024, whereas for NB it is 0.299.

### 4.2. Performance of the Models with a Balanced Dataset

The second part of our experiment deals with a balanced dataset. Table 7 represents the experimental results. In this part, ADA achieves classifications for ACC, SEN, SPEC, F1-Score, and AUC of 96.15%, 0.961, 0.962, 0.961, and 0.966, respectively, which is better than the other models. Similarly, LR attains a poor accuracy of 83.01% and the AUC is 0.704.

In the next phase, we followed the same strategy used earlier for the imbalanced dataset with five-fold CV. The results are shown in Table 8. Among all classifiers, RF achieves an optimal accuracy of 90.47% and SEN, SPEC, F1-score, and AUC of 0.909, 0.999, 0.919, and 0.998, respectively. Conversely, the SVM attained a low accuracy of 81.92% and an AUC of 0.737.

After thorough analysis, the final observations are listed in Table 9 and Table 10. Table 9 shows the optimal classifier performance comparison based on different validation techniques (hold-out and five-fold CV) for the balanced and imbalanced datasets. The best classifiers are ADA (ACC: 96.15%) and RF (ACC: 96.67%) for balanced and imbalanced datasets, respectively, using hold-out CV. Similarly, RF (ACC: 90.47%) and SVM (ACC: 88%) attained maximum efficacy with balanced and imbalanced datasets using five-fold CV. 

On the other hand, Table 10 represents the poor classifier performance comparison based on hold-out and five-fold CV with the balanced and imbalanced datasets. Based on the results, we found that LR and DT attained poor accuracies of 83.01% and 80% with an imbalanced and balanced dataset, respectively, via hold-out CV. Similarly, SVM and NB obtained accuracies of 81.92% and 67% with a balanced and imbalanced dataset, respectively, via five-fold CV. 

### 4.3. Unboxing the Good and Poorly Performed AI Models via SHAP on a Balanced Dataset

We unbox different AI models based on their performance for balanced and imbalanced datasets. In this way, we found four AI models, including ADA and LR, performed best, whereas RF and SVM performed poorly on the balanced dataset. On the other side, four AI systems, including RF and SVM, provided good performance scores for the imbalanced dataset and DT and NB performed poorly. Hence, interpretability must be required to comprehend the reason behind this outcome. Here, we introduced an XAI technique approach that produces trustworthy predictive modeling outcomes. The XAI approach, known as Shapley additive explanations (SHAP), is applied to the black-box classifier and provides a straightforward human interpretation. SHAP favors utilizing various visualizations to highlight the significance of features and how they affect predictions. In SHAP, the y-axis is determined by features and the x-axis by Shapley values. The red color indicates the feature value as high, and blue is for low. The features are ordered according to their importance. 

**Figure 3:**
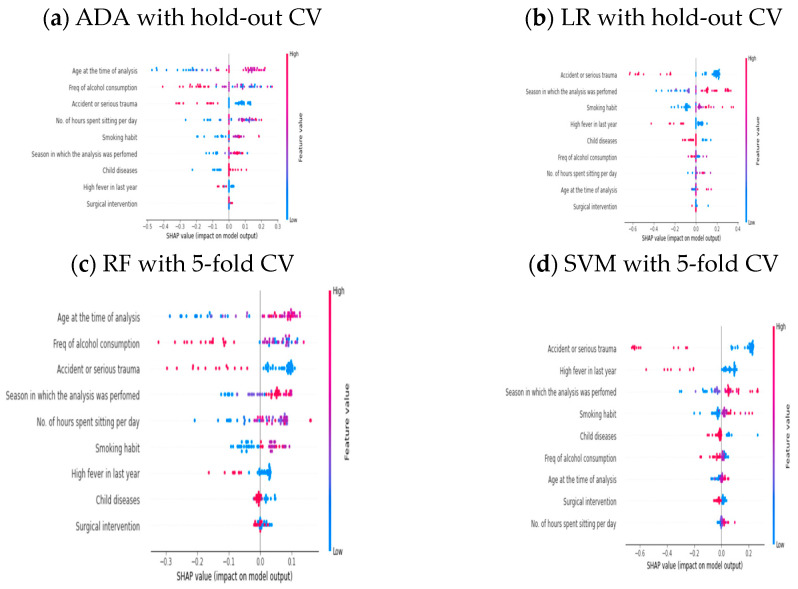
SHAP explanation for balance dataset.

Figure 3a–d show the outcome of the SHAP approach for the existing AI models based on performance using SMOTE where hold-out and five-fold CV are considered. First, we unbox the ADA classifier model, which provides the highest accuracy of 96.15% for hold-out CV. Observing Figure 3a, we found that the $f_{2}$ and $f_{5}$ features are the top and most minor significant feature, respectively, that played a critical role in prediction. Similarly, Figure 3b represents the LR model summary plot, where the $f_{4}\;$ feature is considered a top feature and the $f_{5}\;$ feature has significantly less effect on the LR classifier model. In both cases, the common feature is surgical intervention ( $f_{5}),$ which is the least significant feature. Others feature values are varied and that is why accuracy differs. LR attained 83.01% accuracy, the poor performance for oversampling via hold-out CV. In the next stage, we open another black box such as RF and SVM models. RF achieved an accuracy of 90.47% and SVM attained 81.92% using five-fold CV with oversampling. Figure 3c shows each feature’s role in the RF classifier model on prediction outcome. For the RF model, $f_{2}\;$ is a top feature, whereas $f_{5}\;$ is the last feature according to the Shapley value. When we unbox (see Figure 3d) the SVM model, $f_{4}\;$ is a most impactful feature, whereas $\;f_{9}$ is the last feature. Overall, after comparison of both models, we concluded that the values of each feature from $f_{1}$ to $f_{9}$ are varied. Hence, the prediction outcome differs. 

### 4.4. Unboxing the Good and Poorly Performed AI Models via SHAP on an Imbalanced Dataset

**Figure 4:**
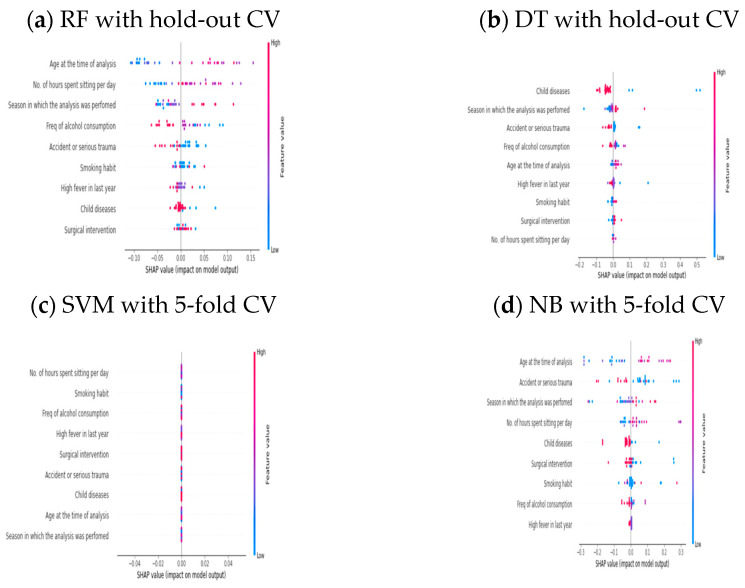
SHAP Explanation for Imbalance Dataset.

After unboxing existing AI models for the balanced dataset, the same strategy was followed to open other existing AI systems for an imbalanced dataset. Table 9 and Table 10 show that DT and RF performed well, whereas the SVM and NB classifier models provided poor prediction outcomes. This categorization has been performed via different CV approaches. We unbox four AI systems, and Figure 4a–d represents all system insights using the Shapley value. Figure 4a shows the global explainability of the RF model, which obtained an accuracy of 96.67% via hold-out CV. According to the SHAP outcome, $f_{2}$ is a top feature among all and $f_{5}$ is identified as the least significant feature. Similarly, when we used SHAP on the DT model it provides the least accuracy of 80%. Figure 4b presents the DT model’s explanation according to the Shapley value of each feature. The same feature, $f_{2}$ , is also identified as the top feature found in RF model. The low impact feature is $f_{6}$ , which is different; the other features’ Shapley values are varied. As a result, low accuracy is identified compared with other AI systems. Figure 4c shows the explanation of the SVM model, which achieved a mean accuracy of 88% via five-fold CV. The top and last features of the SVM model are $f_{9}\;$ and $f_{1}$ , respectively. This accuracy is greater than the other AI systems. Figure 4d shows the NB model explanation, where we observed that the top feature is $f_{3}\;$ and the minor feature is $f_{9}$ for this model prediction. This model achieved a mean accuracy of 67%, which was much less than others.

## 5. Discussion

Few works have been reported in male fertility detection using environmental and behavioral factors. Most studies deal with an imbalanced dataset with missing model interpretation, which motivated our research. We employed the same techniques [8,9,10,11,12,13,14,19] and performance evaluation measures (ACC in %, SEN, SPEC, F1-Score, and AUC) to detect male fertility. Besides this, two more essential steps, data balancing and validation, are considered to build a robust and effective prediction model. We compared all model performance between balanced and imbalanced datasets with two cross-validation schemes (five-fold and hold-out). Our experimental approach is different; thus, direct comparison is not meaningful with existing AI models (see Table 11). Note that all researchers did not use all performance evaluation metrics. We mentioned all values (see Table 5, Table 6, Table 7, Table 8, Table 9 and Table 10), which is helpful for future researchers for better understanding. Additionally, implementing the XAI tool on existing models is another point of attraction and makes our study more special. The utility of this tool is to uncover the model inside and provide a proper explanation. As a result, clinicians can easily understand how AI models work and which features are more impactful for decision making. After the four-stage-based rigorous experimentation, we found that RF, SVM, and ADA (see Table 9) provided good classification accuracy and AUC, whereas LR, DT, and NB performed poorly (see Table 10). Finally, RF is the sole classifier that works well in every situation. At the same time, the SVM classifier achieved a good AUC via five-fold CV with the imbalanced dataset. Conversely, the same classifiers attained poor AUC (0.737) via five-fold CV with the balanced dataset. We primarily focused on robust, stable, and unbiased AI systems; for this reason, we only considered a five-fold-CV-based model with excellent AUC value. In this way, we proposed an effective explainable RF model that achieved AUC and ACC values of 0.998 and 90.47%, respectively. This explainable model achieved a remarkable AUC among seven industry-standard ML models. We also provided the SHAP explanations of existing models that performed well and poorly. For better user understanding, we have provided a bird’s-eye view that implicates each feature’s role in the decision-making process to these black boxes (see Table 12). In Table 12, considering our experiments, we observed that age is the most relevant predictor of male infertility. Moreover, the proposed explainable model provides a reference for clinicians in detecting and preventing male infertility.

To the best of our knowledge, our proposed model provides better prediction results than conventional statistical models. Semen analysis is a cornerstone for predicting male fertility according to the WHO. For this, laboratory tests and/or clinical evaluations are required to examine semen parameters. Typical statistical approaches include regression, correlation, and variance analyses (plus survival analysis) to identify the factors that affect male fertility [42,45,46,47]. However, the type of data is related to sperm videos and seminal data, whereas in this study we have used environmental and lifestyle factors that are not related to laboratory test data. Because of which, our study is different and direct comparison is not applicable with existing conventional statistical models.

## 6. Conclusions

The study reported in this paper has been directed towards the assessment of male fertility via seven existing ML tools, namely, SVM, RF, DT, LR, NB, ADA, and MLP. Based on the performance evaluation, we identified the good and poor classifiers with five-fold and hold-out CV for balanced and imbalanced datasets. Furthermore, we eventually uncovered these models using SHAP; thus, prediction results have enhanced transparency and explainability. After the vivid analysis, we proposed an explainable RF model that can efficiently detect male fertility. The model achieved good accuracy and AUC values of 90.47 and 0.998, respectively. Due to explainability, clinicians can easily understand the impact of each feature on the decision-making process. As a result, real-time implementation and commercialization have become easy. In this current study, the XAI-integrated model for the detection of male fertility is evaluated with limited samples and a single dataset. The future scope of this work predominately focuses on applying other AI algorithms with different sampling-process-based model designs.

## Figures and Tables

**Table 1:** Experimental Dataset [12].

Features. No	Feature’s Name	Values Range (Max–Min)	Normalized
$f_{1}$	Season	winter, spring, summer, and fall	(−1, −0.33, 0.33, 1)
$f_{2}$	Age	18–36	(0, 1)
$f_{3}$	Childhood Disease	yes or no	(0, 1)
$f_{4}$	Accident/Trauma	yes or no	(0, 1)
$f_{5}$	Surgical Interventional	yes or no	(0, 1)
$f_{6}$	High Fever	less than 3 months ago, more than 3 months ago, no	(−1, 0, 1)
$f_{7}$	Alcohol Intake	several times a day, every day, several times in a week, and hardly ever or never	(0, 1)
$f_{8}$	Smoking Habit	never, occasional, and daily	(−1, 0, 1)
$f_{9}$	Sitting Hours/day	1–16	(0, 1)
$f_{10}$	Target Class	normal or altered	(1, 0)

**Table 2:** Statistical overviews of the fertility dataset.

	$f_{1}$	$f_{2}$	$f_{3}$	$f_{4}$	$f_{5}$	$f_{6}$	$f_{7}$	$f_{8}$	$f_{9}$	$f_{10}$
Count	100.000000	100.000000	100.000000	100.000000	100.000000	100.000000	100.000000	100.000000	100.000000	100.000000
Mean	−0.078900	0.669000	0.870000	0.440000	0.510000	0.190000	0.832000	−0.350000	0.406800	1.120000
Std	0.796725	0.121319	0.337998	0.498888	0.502418	0.580752	0.167501	0.808728	0.186395	0.326599
Min	−1.000000	0.500000	0.000000	0.000000	0.000000	−1.000000	−0.200000	−1.000000	0.060000	1.000000
25%	−1.000000	0.560000	1.000000	0.000000	0.000000	0.000000	0.800000	−1.000000	0.250000	1.000000
50%	−0.330000	0.670000	1.000000	0.000000	1.000000	0.000000	0.800000	−1.000000	0.380000	1.000000
75%	1.000000	0.750000	1.000000	1.000000	1.000000	1.000000	1.000000	0.000000	0.500000	1.000000
Max	1.000000	1.000000	1.000000	1.000000	1.000000	1.000000	1.000000	1.000000	1.000000	2.000000

**Table 3:** Correlation values of the dataset.

	$f_{1}$	$f_{2}$	$f_{3}$	$f_{4}$	$f_{5}$	$f_{6}$	$f_{7}$	$f_{8}$	$f_{9}$	$f_{10}$
$f_{1}$	1.000000	0.065410	−0.176509	−0.096274	−0.006210	−0.221818	−0.041290	−0.028085	−0.019021	0.192417
$f_{2}$	0.065410	1.000000	0.080551	0.215958	0.271945	0.120284	−0.247940	0.072581	−0.442452	0.115229
$f_{3}$	−0.176509	0.080551	1.000000	0.162936	−0.140972	0.075645	0.038538	0.090535	−0.147761	−0.040261
$f_{4}$	−0.096274	0.215958	0.162936	1.000000	0.103166	−0.082278	−0.242722	0.110157	0.013122	−0.141346
$f_{5}$	−0.006210	0.271945	−0.140972	0.103166	1.000000	−0.231598	−0.075858	−0.053448	−0.192726	0.054171
$f_{6}$	−0.221818	0.120284	0.075645	−0.082278	−0.231598	1.000000	−0.000831	−0.007527	−0.151091	−0.121421
$f_{7}$	−0.041290	−0.247940	0.038538	−0.242722	−0.075858	−0.000831	1.000000	−0.184926	0.111371	−0.144760
$f_{8}$	−0.028085	0.072581	0.090535	0.110157	−0.053448	−0.007527	−0.184926	1.000000	−0.106007	0.045891
$f_{9}$	−0.019021	−0.442452	−0.147761	0.013122	−0.192726	−0.151091	0.111371	−0.106007	1.000000	0.022964
$f_{10}$	0.192417	0.115229	−0.040261	−0.141346	0.054171	−0.121421	−0.144760	0.045891	0.022964	1.000000

**Table 4:** Handling imbalanced classes using oversampling.

Target Class	Before SMOTE	Total Data Size	After SMOTE	Training Data Size
Normal sperm quality	88	100	88	172
Altered sperm quality	12		84	

**Table 5:** Performance with imbalanced data set using hold-out CV.

Models	Test Set Performances				
	ACC (in%)	SEN	SPEC	F1-Score	AUC
SVM	93.33	0.933	0.091	0.965	0.887
RF	96.67	0.965	0.965	0.982	0.932
DT	80.00	0.956	0.142	0.863	0.648
LR	93.33	0.931	0.098	0.965	0.862
NB	86.66	0.928	0.079	0.928	0.664
ADA	93.33	0.964	0.599	0.964	0.908
MLP	93.30	0.965	0.548	0.964	0.746

**Table 6:** Average performance with imbalanced data set using 5-fold CV.

Models	Test Set Performances					
	ACC (in%)	SEN	SPEC	F1-Score	STD	AUC
SVM	88.00	1.000	0.997	0.965	0.024	0.959
RF	87.99	0.943	0.711	0.982	0.070	0.720
DT	74.99	0.817	0.670	0.884	0.141	0.625
LR	88.00	1.000	0.817	0.965	0.024	0.910
NB	67.00	0.734	0.570	0.928	0.299	0.500
MLP	80.99	0.809	0.742	0.964	0.050	0.694
ADA	80.00	0.884	0.933	0.964	0.141	0.839

**Table 7:** Performance with the balanced data set using hold-out CV.

Methods	Test Set Performances				
	ACC (in%)	SEN	SPEC	F1-Score	AUC
SVM	84.90	0.951	0.789	0.826	0.807
RF	92.45	0.958	0.838	0.919	0.940
DT	84.09	0.909	0.806	0.833	0.798
LR	83.01	0.905	0.781	0.808	0.704
NB	88.67	0.954	0.839	0.875	0.749
MLP	83.01	0.904	0.781	0.808	0.801
ADA	96.15	0.9615	0.962	0.961	0.966

**Table 8:** Performance with balanced data set using five-fold CV.

Models	Test Set Performances					
	ACC (in%)	SEN	SPEC	F1-Score	STD	AUC
SVM	81.92	0.706	0.808	0.826	0.097	0.737
RF	90.47	0.909	0.999	0.919	0.133	0.998
DT	85.87	0.830	0.897	0.833	0.070	0.741
LR	82.47	0.752	0.879	0.809	0.088	0.763
NB	85.87	0.797	0.863	0.876	0.108	0.750
MLP	85.90	0.762	0.848	0.809	0.113	0.796
ADA	87.01	0.826	0.817	0.962	0.096	0.893

**Table 9:** Comparison: optimal accuracy between balanced and imbalanced datasets with different CVs.

Dataset	Models	CV Schemes	Test ACC (in %)	Test AUC
Balanced	ADA	Hold-out	96.15	0.966
	RF	5-fold	90.47	0.998
Imbalanced	RF	Hold-out	96.67	0.932
	SVM	5-fold	88.00	0.959

**Table 10:** Comparison: poor accuracy between balanced and imbalanced datasets with different CVs.

Dataset	Methods	CV Schemes	Test ACC (in %)	Test AUC
Balanced	LR	Hold-out	83.01	0.774
	SVM	5-fold	81.92	0.737
Imbalanced	DT	Hold-out	80.00	0.648
	NB	5-fold	67.00	0.500

**Table 11:** Comparison.

Models	ACC (in %)	SEN	SPEC	F1-Score	AUC	Remarks
SVM, MLP, and DT [7]	86, 86 and 84 (SC), 69, 69, 67 (SM)	94.0, 97.0, 96.0 (SC) 72.0, 73.0, 71.0 (SM)	40.0, 20.0, 13.0 (SC) 25.0, 12.0, 12.0 (SM)	-	-	SC = Sperm morphology. SM = Sperm concentration individually measured
DT, MLP, SVM, SVM-PSO, and NB [8]	89, 92, 91, 94, and 89	-	-	-	73.5, 72.8, 75.8, 93.2, and 85.0	Feature selection applied
MLP, NB, DT, and SVM [9]	93.3, 73.10, 83.82, and 80.88	-	-	-	93.3, 81.0, 85.8, 88.2	Optimize MLP
MLP [10]	90, and 82	95.4 and 89.2	50 and 43.7	-	-	-
NB, NN, LR, and Fuzzy C-means [11]	-	-	-	-	75.1, 78.2, 46.6, 69.0	Filtering applied
DT and NB [12]	61.36 and 88.63	-	-	-	-	
MLP, SVM, DT, and FRBF [13]	69.0, 69.0, 67, and 90	72.0, 73.0, 71.0, and 92.0	25.0, 12.0, 12.0, and 50.0	-	-	
ANN and NB [14]	97	-	-	-	-	Testing accuracy not reported
NB [15]	87.75	-	-	-	-	-
ANN, ANN-GA, DT, SVM, and ANN-SWA [16]	90, 95, 88, 95, and 99.96	92.0, 97.0, 83.0, 97.0, and 99.0	71.0, 70.0, 82.0, 72.0, and 99.0	-	-	
J48, SMO, NB, and lazy IBK [17]	-	-	-	-	-	Classification and clustering performed
SVM, AdaBoost, and BPNN [18]	81.6, 95.1, and 91.6	-	-	91.3, 97.2, and 91.6	-	ELSMOTE is used
DT, Bagged DT, RF, and ET [19]	78.80, 88.12, 89.07, and 90.02	-	-	66 (ET)	-	Not reported all models of AUC
KNN [20]	90.00	-	-	-	85.7	-
MLP, SVM, NB, RF, KNN, and FFNN-LBAAA [21]	81, 72, 87.2, 91.3, 84.9, and 97.5	75.0, 69.0, 90.0, 92.0, 81.0, and 93.0	87.0, 74.0, 85.0, 90.0, 89.0, and 100	80.0, 71.3,87.8,91.5,84.5, and 96.6	81.0, 72.0, 87.0, 91.0, 85.0, and 97.0	SMOTE is used
XGB [22]	93.22	95.0	95.0	-	98.0	SMOTE and XAI tools are used
RF _XAI *	90.47	90.98	99.99	91.99	99.98	5-fold CV, SMOTE, and SHAP used

* Training and validation AUCs are documented in Appendix A.

**Table 12:** Features impact on an AI model’s decision making via SHAP.

Performance	Methods	CV Schemes	Feature’s Role
Good	ADA-SMOTE	Hold-out	$f_{2,}f_{7}$ , $f_{4}$ , $f_{9},\;f_{8},\;f_{1},\;f_{3},\;f_{6},\;f_{5}$
	RF-SMOTE	Five-fold	$f_{2,}f_{7}$ , $f_{4}$ , $f_{1},\;f_{9},\;f_{8},\;f_{6},\;f_{3},\;f_{5}$
	RF	Hold-out	$f_{2,}f_{9}$ , $f_{1}$ , $f_{7},\;f_{4},\;f_{8},\;f_{6},\;f_{3},\;f_{5}$
	SVM	Five-fold	$f_{9,}f_{8}$ , $f_{7}$ , $f_{6},\;f_{5},\;f_{4},\;f_{3},\;f_{2},\;f_{1}$
Poor	LR-SMOTE	Hold-out	$f_{4,}f_{1}$ , $f_{8}$ , $f_{6},\;f_{3},\;f_{7},\;f_{9},\;f_{2},\;f_{5}$
	SVM-SMOTE	Five-fold	$f_{4,}f_{6}$ , $f_{1}$ , $f_{8},\;f_{4},\;f_{7},\;f_{2},\;f_{5},\;f_{9}$
	DT	Hold-out	$f_{2,}f_{4}$ , $f_{1}$ , $f_{9},\;f_{3},\;f_{5},\;f_{8},\;f_{7},\;f_{6}$
	NB	Five-fold	$f_{3,}f_{1}$ , $f_{4}$ , $f_{7},\;f_{2},\;f_{6},\;f_{8},\;f_{5},\;f_{9}$

## Data Availability

https://archive.ics.uci.edu/ml/datasets/Fertility (accessed on 8 January 2022).

## Abbreviations

$t_{p}$	True Positive
$t_{n}$	True Negative
$f_{p}$	False Positive
$f_{n}$	False Negative
TPR	True Positive Rate
FPR	False Positive Rate

## Appendix A

The training and validation AUCs of the proposed RF-SHAP model are summarized in Table A1.

**Table A1:** Performance of RF-SHAP model.

Dataset	Methods	AUCs	
		Training	Validation
Balanced	5-fold CV	1.0	0.97
